# Corrosion-Related Failures in Modular Total Knee Arthroplasty: A Narrative Review of Rare Published Clinical Cases

**DOI:** 10.2106/JBJS.OA.26.00005

**Published:** 2026-05-12

**Authors:** Krithi Pachipala, Amy Z. Lu, Aymen Alqazzaz, Joshua J. Jacobs, Lisa Follett, H. John Cooper

**Affiliations:** 1 Department of Orthopaedic Surgery, Columbia University Medical Center, New York, New York; 2 Weill Cornell Medicine, New York, New York; 3 Department of Orthopaedic Surgery, Rush University Medical Center, Chicago, Illinois

## Abstract

» Corrosion at modular junctions is a well-known cause of failure in primary and revision total hip arthroplasty, but it is underrecognized in modular total knee arthroplasty (TKA) despite the potential to cause clinically significant implant failure.

» Although descriptions vary throughout case reports, mechanically assisted crevice corrosion (MACC) appears to be the underlying failure mechanism at these modular TKA junctions, as is seen in femoral component modularity in total hip arthroplasty (THA).

» Adverse local tissue reactions (ALTR) and fatigue fractures can occur even in well-fixed TKA implants and can be difficult to detect clinically or radiographically.

» The femoral component in constrained or rotating-hinge designs may be at higher risk for corrosion-related complications.

» An appropriate index of suspicion, improved surveillance protocols, advanced imaging, and design innovations are needed to aid diagnosis, mitigate future failures, and guide revision strategies.

## Introduction

Modular implant designs are commonly used in revision total knee arthroplasty (rTKA) to address bone loss and soft tissue deficiency. Each modular junction introduces a new interface susceptible to tribocorrosion, a combination of mechanical wear and electrochemical degradation. Although corrosion is well described in total hip arthroplasty (THA), it is underrecognized in TKA^1^.

Recent cases describe corrosion most commonly between the femoral component and modular stems and sleeves. These failures involve mechanically-assisted crevice corrosion (MACC), adverse local tissue reactions (ALTR), and implant loosening and fatigue fractures. These findings raise concern about the biological and mechanical consequences of MACC in modern modular TKA^2-7^.

This review highlights clinical cases of corrosion-associated TKA failure (Table I) and retrieval studies (Table II) to inform surgeons, researchers, and manufacturers about corrosion risks in modular TKA systems and guide early recognition and mitigation.

**TABLE I:** Case Reports of Clinical Failures from Corrosion in Total Knee Arthroplasty

Study	No. of Patients	Implant Design	Component	Modular Junction	Implant Brand & System	Corrosion Modes Reported
Ekweariri et al.^2^	1	Varus-valgus constrained TKA	Femur	Ti stem and CoCr femoral taper junction (Ti–CoCr junction)	Zimmer NexGen Legacy Constrained Condylar Knee	Taper, fretting, galvanic, metallosis, Co:Cr = 2:1
McMaster and Patel^3^	1	Rotating platform hinged revision TKA	Femur	CoCrMo femoral stem extension and CoCrMo distal femoral component Morse taper junction	DePuy Limb Preservation System	Crevice, taper, fretting/passive, taper, ALTR, ↑ Co
Rako et al.^4^	1	Modular, segmental distal femoral replacement	Femur	(1) CoCr distal femoral component to CoCr extension piece and (2) Ti femoral stem to CoCr extension piece	Stryker Global Modular Replacement System	Taper corrosion (CoCr–Ti), ALTR, Histologic ALVAL, ↑ Co/Cr
Holland et al.^5^	1	Modular revision component with posterior-stabilized nonconstrained liner TKA	Femur	CoCr femoral condylar taper to CoCr stem taper (bolt-compressed Morse taper)	DePuy Synthes SIGMA Revision Knee	Intergranular, chemical corrosion, ALTR, ↑ Co/Cr
Yeramosu et al.^6^	1	Varus-valgus constrained TKA	Femur	CoCr femoral condylar component and Ti femoral stem connected by Morse taper (adapter bolt)—fracture at Morse-taper adapter bolt junction	Press Fit DePuy Synthes SIGMA Total Condylar 3 knee	Taper corrosion, fatigue fracture, fretting, corrosion, ALTR
Christiner et al.^7^	1	Varus-valgus constrained TKA	Femur	Ti femoral stem and CoCrMo metaphyseal sleeve taper-lock junction—fracture at sleeve-stem junction	DePuy Synthes Low Contact Stress Revision Knee	Fretting, crevice corrosion, fatigue, MACC
Rainey et al.^8^	4	Revision TKA with metaphyseal sleeves	Femur and tibia	CoCr femoral/tibial component male trunnion—Ti metaphyseal sleeve female bore (machined taper junction)	Press Fit DePuy Synthes SIGMA Total Condylar 3 knee	MACC, fretting corrosion, metallosis, pseudotumor, elevated serum/synovial Co/Cr

ALTR = adverse local tissue reaction, CoCr = cobalt–chromium, Ti = titanium, TKA = total knee arthroplasty, and MACC = mechanically assisted crevice corrosion.

Published case reports describing clinical failures of total knee arthroplasty (TKA) associated with corrosion at modular metal junctions. Reported corrosion mechanisms include mechanically assisted crevice corrosion (MACC), fretting corrosion, galvanic corrosion, chemical and intergranular corrosion, and taper-related corrosion. Adverse local tissue reactions (ALTR) and serum metal ion elevations are included when reported. Implant design terminology reflects the original authors’ descriptions. Corrosion mechanisms and implant junctions are summarized as reported in each source and were not independently reclassified by the authors.

**TABLE II:** Retrieval Studies and Systematic Reviews of Corrosion in Modular Total Knee Arthroplasty

Study	Type	# implant	Implant Design	Corrosion Modes Reported
Kurtz et al.^9^	Retrieval	50 Ti baseplate-stem pairs	Ti–Ti tibial baseplate–stem taper junction	MACC, crevice corrosion, selective dissolution, pitting
Aslani et al.^10^	Retrieval	17 femoral, 15 tibial	Metaphyseal sleeves with mixed-alloy modular taper junctions (CoCrMo–Ti-6Al-4V)	MACC, pitting, etching, oxide debris
Arnholt et al.^11^	Retrieval	52 femoral components	CoCr	ICIC, third-body wear, cement interface corrosion, metal-on-metal wear; MACC in modular components
Cerquiglini et al.^12^	Retrieval	28 femoral, 9 tibial	Polished CoCr femoral and tibial components (CR and PS designs)	ICIC
Spece et al.^13^	Retrieval	166 modular junctions	Modular femoral and tibial conical taper junctions (multiple designs)	Fretting, MACC
Martin et al.^14^	Retrieval	22 implants	Axial- and radial-screw modular taper junctions (Zimmer NexGen; femoral and tibial)	Fretting corrosion with preferential material loss in mixed-metal (CoCr–Ti) junctions
Luetzner et al.^15^	Clinical metal ion study	41 TKAs	Cemented, unconstrained CoCrMo TKA (no patellar resurfacing)	Elevated serum Co/Cr levels (no retrieval or surface corrosion analysis)
Engh et al.^16^	Systematic review of revision outcomes (THA and TKA)	9 studies (THA/TKA)	Mixed arthroplasty designs (predominantly THA)	Not applicable (review of clinical treatment outcomes)
Berry et al.^1^	Narrative clinical review	—	Multiple arthroplasty designs (THA and TKA)	Conceptual framework for polyethylene wear, metal on metal wear, and taper tribocorrosion
Terhune et al.^17^	Retrieval	82 revision TKAs	Modular revision TKA stem–component junctions (tapered vs. threaded designs)	Fretting corrosion, fretting wear, dark deposits
Christiner et al.^18^	Retrieval	13 revision TKAs	Threaded taper modular junctions (Stryker Triathlon TS; predominantly CoCr)	Fretting corrosion, MACC, cobalt ion dispersion, ALTR (including ALVAL)

CoCr = cobalt–chromium, ICIC = inflammatory cell–induced corrosion, THA = total hip arthroplasty, TKA = total knee arthroplasty, MACC = mechanically assisted crevice corrosion, and Ti = titanium.

Retrieval studies and systematic reviews evaluating corrosion in modular total knee arthroplasty (TKA). Retrieval studies summarize corrosion mechanisms identified at modular metal junctions, including mechanically assisted crevice corrosion (MACC), fretting corrosion, and inflammatory cell–induced corrosion (ICIC). Systematic and narrative reviews are included to provide clinical and conceptual context regarding tribocorrosion mechanisms and management strategies. Corrosion modes, implant designs, and junction types are reported as described in the original studies.

## Insights from TKA Retrieval Studies

Retrieval studies published between 2007 and 2025 describe corrosion at modular TKA junctions; although descriptions of the findings vary, these likely represent different descriptions of similar pathology rather than different forms of failure.

MACC was the most frequently reported failure mechanism, involving micromotion and electrochemical attack within crevices, such as taper junctions between sleeves and stems^9,10,13^. MACC has been identified in both modular femoral and tibial taper interfaces, particularly where titanium (Ti) stems mate with cobalt-chromium-molybdenum (CoCrMo) components^9,14^. Although tibial corrosion is less prevalent than femoral corrosion, it may occur earlier and with greater severity^18^. Characteristic damage includes oxide staining, pitting, and fretting scars, observed even in well-fixed components, suggesting that minimal micromotion may disrupt passive oxide layers and initiate degradation^11^. In addition, it is associated with both local ALTR and systemic metal ion elevation^2,3,15^.

Within this tribocorrosion spectrum, fretting is described as a component of MACC or as a related but distinct manifestation of cyclic micromotion, manifesting with surface wear and deposition of dark oxide debris and occurring at Morse tapers, stem-adapter junctions, and offset components^7,9,13,18^. Fretting often coexists with MACC but can occur independently in areas of poor engagement or suboptimal fit^9,14^.

Other corrosion modes reported in retrievals, which likely reflect an incomplete understanding of the pathology, include inflammatory cell-induced corrosion (ICIC)^11,12^, galvanic (mixed-metal) corrosion, cement-metal interface corrosion, third-body corrosion, and passive crevice corrosion^1,7,12-14,19-21^.

THA retrievals suggest that most corrosion-related failures fall within the tribocorrosion continuum, dominated by MACC and fretting, occasionally compounded by biologic corrosion (ICIC)^22-24^. This pattern likely extends to modular TKA systems, and reported modes of failure such as galvanic, passive crevice, or cement-interface corrosion may occur, but material loss is primarily driven by MACC^1,13,14,19-21^.

## Insights from Clinical Case Reports

To date, the clinical literature includes 6 case reports and a 4-case series describing corrosion-related implant failure in modular TKA.

### Case 1

A 77-year-old man with NexGen Legacy varus-valgus Constrained Condylar Knee (Zimmer) presented 13.5 years after primary TKA with posterior knee pain and an effusion^2^. The implant included a Ti femoral stem coupled to a CoCrMo femoral component at a modular taper junction. Synovial fluid demonstrated elevated leukocytes, and radiographs revealed subtle tibial radiolucency.

At revision, copious gray fluid and extensive black/gray hypertrophic synovitis were present. The femoral component was loose, with significant distal femoral bone loss. Severe corrosion at the Ti-CoCrMo junction was consistent with MACC, along with metallosis and ALTR. The well-fixed tibial component was retained. Reconstruction involved a distal femoral cone and new femoral component. Postoperative metal ions were elevated (Co 1.1, Cr 0.7), yielding a 2:1 CoCr ratio. The patient was pain-free 4 months postoperatively.

This case demonstrates that modular TKA designs are susceptible to MACC at taper junctions, which can present as a delayed failure. Preoperative metal ion testing with CT or metal artifact suppression sequence magnetic resonance imaging (MRI) may have aided in earlier diagnosis.

### Case 2

A 65-year-old man presented 20 months after rTKA with progressive swelling, stiffness, pain, and erythema with a DePuy Limb Preservation System rotating platform hinged prosthesis (Johnson & Johnson [J&J])^3^. Erythrocyte sedimentation rate (ESR) (122), c-reactive protein (CRP) (162), and Serum Co (30) were elevated, with undetectable Cr. Aspiration showed low leukocyte counts and negative cultures.

At revision, clear fluid and a thickened avascular capsule were noted with well-fixed components. Black debris was present at the Morse taper between the CoCrMo femoral component and the CoCrMo femoral stem. Corrosion discoloration and etching were evident on both surfaces. Energy-dispersive x-ray spectroscopy confirmed oxygen, Cr, and Mo peaks, consistent with products of the CoCrMo alloy.

The distal femoral component was exchanged for another CoCrMo part due to a lack of alternative hinge designs. At 8 months, serum Co (2.3) and Cr (0.7) had normalized, and he remained symptom-free.

Taper corrosion in hinged modular TKAs may present with pain, soft tissue changes, and elevated serum Co despite normal radiographs. Although both components were CoCrMo, corrosion is driven by micromotion and crevice electrochemistry rather than sliding articulation and therefore does not represent a metal-on-metal bearing surface failure^11^. The highly constrained hinged construct and the long stem likely increased bending and torsional stresses, amplifying micromotion and disrupting the protective oxide layer over time^11^.

### Case 3

A 69-year-old woman with distal femoral replacement (DFR) (Global Modular Replacement System; Stryker) and proximal tibial replacement presented with months of pain and fluctuance of the proximal tibia^4^. Radiographs demonstrated progressive osteolysis.

Workup showed elevated ESR (39), CRP (14), Co (5.3), and Cr (1.6). Two aspirations yielded brown fluid with markedly elevated leukocyte counts; cultures remained negative. Although the 2018 periprosthetic joint infection (PJI) criteria score was 6, raising concern for infection, elevated metal levels and the aspirate’s appearance prompted suspicion for corrosion. Revision revealed blackened capsule, dark fluid, tissue necrosis, and bone loss of the proximal tibia and distal femur. Severe corrosion was identified at 2 femoral taper junctions (Ti stem–CoCrMo extension and CoCrMo extension–CoCrMo femoral component). New modular CoCrMo femoral components were implanted onto the well-fixed Ti stem. The well-fixed tibial stem was retained.

Histopathology showed extensive necrosis and dense histiocytic infiltration, consistent with ALTR secondary to MACC. At 15 months, the patient reported sustained pain relief, and radiographs revealed well-fixed implants with evidence of bone remodeling.

This first report of ALTR from taper corrosion at 2 femoral modular junctions underscores how ALTR can mimic infection. While metal artifact reduction sequence MRI was not obtained, it can aid soft-tissue assessment in suspected taper corrosion. Management mirrored THA taper protocols: retain well-fixed stem, manually clean rather than aggressively abrade the taper (to avoid altering cone geometry/roughness), and exchange damaged modular CoCrMo parts.

### Case 4

A 61-year-old man presented 13 years after rTKA with cemented posterior-stabilized DePuy Synthes SIGMA (J&J) with years of progressive pain, swelling, and instability^5^. The modular construct consisted of a CoCrMo femoral condylar component attached to a CoCrMo stem extension through a Morse taper compressed by an intercondylar locking bolt. Serum metal levels were elevated (Co 7.3; Cr 4.6). Radiographs showed a subtle radiolucent line at the condylar-stem interface.

At revision, there was metallic gray synovitis. The patellar and tibial components were well fixed; however, the femoral condylar component was loose at the modular junction. Retrieval analysis demonstrated extensive corrosion and material loss involving the CoCrMo femoral condylar and stem extension Morse taper interface, with black corrosion debris present. The intercondylar locking bolt was intact, with debris noted along the threaded portion but without fracture. The tibial polyethylene insert exhibited delamination and pitting.

Reconstruction included a press-fit trabecular metal femoral cone and a cemented revision femoral component and stem. Synovial pathology showed diffuse histiocytic infiltration with metallic debris. Postoperatively, metal levels declined, and symptoms improved at 1 year. Although described as “chemical corrosion,” these findings are more consistent with mechanically-assisted intergranular corrosion within the spectrum of MACC, driven by micromotion and grain boundary attack.

### Case 5

A 65-year-old woman (body mass index [BMI] 37.8) with a prior varus-valgus constrained Press Fit Condylar DePuy Synthes Sigma TC3 rTKA (J&J) composed of a Ti femoral stem and CoCrMo condylar component joined by a Morse taper and adapter bolt presented with progressive pain and swelling^6^. MRI identified a fracture at the stem–taper/adapter-bolt junction that had not been evident on x-rays.

At revision, the adapter bolt was fractured with a fragment lodged in the Morse taper, and the femoral component was grossly unstable. Corrosion at the taper/bolt interface was consistent with MACC contributing to the adapter’s fatigue failure. Reconstruction proceeded with DePuy S-ROM rotating-hinged knee, retaining the stable tibial component. Postoperatively, she had sustained pain relief and functional improvement.

### Case 6

A 66-year-old highly active man (BMI 32) with a prior varus-valgus constrained TKA using a DePuy Low Contact Stress (LCS; Johnson & Johnson) system constructed with a CoCrMo femoral metaphyseal sleeve mated to a cemented Ti femoral stem presented years later with progressive pain and swelling^7^. Radiographs demonstrated osteolysis without hardware dissociation, but CT revealed a fatigue fracture at the femoral sleeve–stem modular junction with bone loss.

At revision, gray fluid, metallosis, and hypertrophic stained synovium were present. The femoral component was loose, with fracture at the sleeve-stem interface. Retrieval demonstrated fretting and crevice corrosion with pitting at the modular junction; failure initiated at a stem diameter step-down, a geometric stress riser that facilitated MACC-related fatigue failure. There was substantial condylar/metaphyseal bone loss, but the tibial component was well fixed and retained. Following extensive debridement of stained synovium and removal of corrosion products, the femur was reconstructed with metaphyseal support (cone/sleeve) and a new femoral component/stem. Symptoms resolved postoperatively.

This report highlights a sleeve-stem modular junction as a corrosion-prone weak point in constrained TKAs, evident in an obese and active individual.

### Rainey et al. Case Series

Rainey et al. reported 4 cases of rTKAs with metaphyseal sleeves in which metallosis was attributed to MACC at the machined trunnion-bore taper interfaces between CoCrMo components and Ti sleeves^8^. Three junction patterns were described: (1) femoral component trunnion-femoral sleeve bore, (2) tibial baseplate stem/trunnion-tibial sleeve bore, and (3) a hinged femoral component trunnion mated to a retained, well-fixed femoral sleeve with progressive bone loss despite apparently well-fixed components.

### Case 7

An 87-year-old developed knee pain after rTKA (Sigma TC3)^8^. Infection markers were low, but synovial Co was elevated (30). Bone scan showed focal uptake at the femoral component. At revision, the bore of the femoral sleeve showed mechanical etching/scoring with corrosion debris, and the femoral taper showed wear and mild corrosion.

### Case 8

A 64-year-old with bilateral rTKAs (Sigma TC3 with sleeves) developed progressive pain and stiffness^8^. Aspirates showed elevated synovial metal ions in both knees. At staged revisions, the right knee had corrosion at the femoral trunnion–sleeve bore interface, and the left had corrosion at the tibial tray-sleeve interface with a loose tray and retained sleeve. Both were revised to hinged constructs with metaphyseal cones.

### Case 9

A 65-year-old with arthrogryposis had a well-fixed femoral sleeve later mated to a hinged femoral component and developed recurrent effusions and progressive anterior cortical bone loss despite well-fixed components^8^. Serum Co/Cr elevated; synovial Co reported extremely high. Intraoperatively, the femoral trunnion grooves and retained sleeve bore contained black corrosion debris; revision required removal of the femoral sleeve/stem and conversion to a segmental DFR due to bone loss.

### Case 10

A 66-year-old presented with pain and a large medial soft tissue mass; imaging showed medial tibial plateau erosion and a fluid collection^8^. Intraoperatively, a pseudotumor-like mass was decompressed, and corrosion debris was found at the distal end of the tibial female bore, with additional femoral trunnion/sleeve corrosion. Reimplantation used short, cemented stems/cones and an oxidized zirconium femoral component.

## Diagnostic and Design Implications

Retrievals discuss high prevalence of corrosion across both femoral and tibial components and reported that corrosion severity varied according to component location (although reported clinical failures frequently involve femoral modular junctions), modular junction design (conical versus threaded), and patient body weight and activity level^18^. Corrosion was identified in 54% of modular taper junctions in revision TKAs that were performed for infection or aseptic loosening rather than suspected corrosion^17^. The discrepancy between frequent retrieval corrosion and few reported clinical failures suggests a progressive, threshold-dependent process rather than an immediately symptomatic event. Corrosion can occur in well-fixed components and remain subclinical before presenting as metallosis, ALTR, osteolysis, or mechanical failure. Taper geometry, material pairing, constraint level, and stem length likely influence severity and presentation. Implant design, material combinations, patient activity, and loading conditions likely influence whether corrosion becomes clinically apparent.

Several patterns emerged:

- Modular taper junctions are particularly vulnerable to MACC and fretting, particularly in rotating-hinge and constrained designs. These sites are often embedded within bone and implant architecture, making early detection challenging without blood metal levels, advanced imaging, or intraoperative visualization^3,6,13^.
- Stem-sleeve junctions and metaphyseal adapters, while designed to enhance fixation, can be sites of MACC when conditions such as micromotion, fluid entrapment, and reduced contact area are present^9,10,13^. Severe corrosion may be more common in tapered than threaded junctions^17^.
- Mixed-metal pairings (e.g., Ti stems with CoCrMo sleeves or condyles) demonstrate more severe fretting and MACC, likely reflecting differences in material hardness, flexural rigidity (elastic incompatibility), and oxide stability rather than simple galvanic corrosion^4,12,13^.
- Corrosion-related fatigue fractures highlight a mechanical consequence of tribocorrosion and are often preceded by nonspecific symptoms or subtle radiographic findings^6,7,13^.
- Many cases of corrosion involve patients who are either overweight or reported high activity levels, suggesting a potential role of mechanical loading^18^. It is noted patient weight correlates with increased corrosion at threaded taper junctions.

Taken together, these findings suggest that corrosion in modular TKA may be underrecognized due to nonspecific symptoms, normal radiographs, well-fixed components, and limited familiarity with corrosion pathology, necessitating improved surveillance strategies, such as:

- Serum metal level screening in patients with unexplained pain who have modular TKA implants^3^.
- Serum Co and Cr levels may identify high-risk patients^15^. No validated threshold exists for TKA, but data from metal-on-polyethylene THA suggest that levels ≥1 ng/mL (1 ppb) may be associated with taper-related ALTRs and should prompt further evaluation in symptomatic patients^25,26^.
- Early use of advanced imaging is recommended when symptoms are unexplained^7^. Metal suppression MRI and ultrasound may detect ALTR in TKA, similar to metal-on-metal hips^1,16^. If plain radiographs are inconclusive, cross-sectional imaging with CT or MRI should be considered to assess for underlying fatigue fractures.
- More robust intraoperative inspection of modular junctions whenever possible, even when components appear well-fixed^4^.

Recognizing these patterns and incorporating corrosion into diagnostic workflows may facilitate earlier revision and reduce long-term complications.

### Treatment Strategies and Outcomes

Corrosion-related complications in TKA are challenging due to subtle preoperative findings and often extensive intraoperative damage. Management depends on corrosion location, implant stability, soft tissue involvement, and patient factors. Revision is the primary treatment, and the extent of component exchange depends on the failure mechanism.

### Surgical Interventions

As in THA cases, modular component exchange may be considered when the corrosion is localized and the stem remains well-fixed. Taper corrosion in the DFR was managed by exchanging modular components while retaining the Ti stem^4^. This approach carries risk if the taper is inadequately debrided or components are incompatible, requiring meticulous cleaning and reassembly.

Extensive corrosion, metallosis, soft tissue necrosis, or poor taper engagement typically necessitate complete revision with aggressive debridement^3,4^. In the ALTR case, complete removal of the stem and Morse taper was necessary due to extensive corrosion debris^3^. Cases involving metaphyseal sleeves or highly ingrown stems pose additional technical challenges, as removal risks further bone loss or fracture^7,10^. Surgeons must balance the morbidity of full revision against the risk of recurrent corrosion with partial exchange.

Preventing recurrence requires careful implant selection and surgical technique. Although monolithic and ceramic designs reduce corrosion risk in THA, their role in revision TKA is limited. In constrained or hinged constructs, junctions should be clean and dry before assembly, with proper taper alignment and impaction^1,9,16^.

### Outcomes After Revision

Short-term outcomes following revision for corrosion-related failure are generally favorable, with pain relief, improved function, and normalization of serum metal levels^3,5^. However, long-term data remains limited and recurrence remains a concern, particularly when modular designs are reused and when risk factors persist^4,13^.

### Future Directions and Research Needs

Reducing corrosion in modular TKA requires optimization of implant design and materials. Modifiable factors include taper geometry, contact area, modular junction tolerances, metallurgical processing, and surface finish. Retrieval studies show these features significantly influence susceptibility to MACC and fretting, particularly in mixed-metal junctions^13,27^.

Alternative materials and coatings may further mitigate corrosion. Although ceramic heads with Ti sleeves have reduced trunnion corrosion in THA, their application in TKA remains limited due to different mechanical demands^1^. Manufacturers should consider standardizing taper dimensions to facilitate cross-systems compatibility and improve quality of machining and finishing processes. Preclinical testing must include tribocorrosion models that replicate the in vivo tribochemical environment, including joint fluid, synovial proteins, and cyclic loading^1^.

## Conclusion

Corrosion in TKA, once considered a rare, is being recognized as a contributor to implant failure^3,4,13,14^. Tribocorrosion at modular junctions, such as metaphyseal sleeves, stem interfaces, and femoral or tibial tapers, can cause mechanical degradation and biologic complications, including ALTR, elevated metal ions, soft tissue damage and implant fracture^5,7,10,13^.

Knowledge gaps remain in diagnostic thresholds, monitoring strategies, and implant-specific risk profiles. Addressing this will require integration of clinical data, materials science, and biologic investigation to inform implant design, guide surgical decision-making, and support early identification of at-risk patients^1,10,12^.

As modular implant use expands in TKA, tribocorrosion must be recognized as a relevant and potentially preventable failure mechanism. Greater awareness among surgeons, improved implant design, and robust post-market surveillance are essential to improving long-term outcomes.
